# Clinical efficacy of drug-eluting stents in the treatment of vertebral artery stenosis and analysis of factors contributing to stent restenosis

**DOI:** 10.3389/fneur.2026.1793654

**Published:** 2026-04-09

**Authors:** Xiuping Wei, Zhi Ma, Huimin Wu, Haibo Jiang, Wenya Lan, Lili Xu, Mingyang Du, Hui Cao, Xin Wang, Feng Qiu

**Affiliations:** 1 Cerebrovascular Disease Center, Nanjing Brain Hospital Affiliated to Nanjing Medical University, Nanjing, China; 2 Department of Neurology, Nanjing Brain Hospital Affiliated to Nanjing Medical University, Nanjing, China

**Keywords:** drug-eluting stents, stenosis, stent restenosis, stroke, vertebral artery

## Abstract

**Background:**

Vertebral artery stenosis, especially at the ostium, is a significant cause of posterior circulation ischemic events. Although drug-eluting stents (DESs) have demonstrated superior efficacy in reducing restenosis compared to bare-metal stents (BMSs), the specific incidence and predictors of in-stent restenosis (ISR) following DES implantation in patients with ostial vertebral artery stenosis (OVAS) remain inadequately investigated.

**Method:**

This single-center retrospective study evaluated 446 patients who underwent DES implantation for OVAS between January 2022 and July 2025. Comprehensive clinical, imaging, pharmacogenomic, and thromboelastography (TEG) data were collected. ISR was defined as ≥50% luminal narrowing within or at its margins during follow-up. Risk factors for ISR were analyzed using univariate and multivariate logistic regression models. Receiver operating characteristic (ROC) curve was used to analyze the predictive value of age, smoking, hypertension, stent tortuosity, CYP2C19 poor metabolizer status and TEG parameters for patients with ISR.

**Results:**

Of the 223 patients with complete follow-up data, 28 (12.56%) developed ISR within one year. Univariate analysis identified age, hypertension, smoking, stent tortuosity, CYP2C19 poor metabolizer status and elevated TEG parameters (*α*-Angle and MA value) as significantly associated with ISR. Multivariate analysis confirmed that α-Angle (OR 1.215, 95% CI 1.025–1.441, *p* = 0.025) and MA value (OR 1.249, 95% CI 1.025–1.522, *p* = 0.027) were independent predictors of ISR. ROC analysis demonstrated excellent predictive performance of *α*-Angle (AUC 0.826) and MA (AUC 0.814) for ISR risk stratification.

**Conclusion:**

ISR remains a significant clinical challenge after DES implantation for OVAS. A combination of clinical, genetic and coagulation factors contributes to its development. TEG parameters, particularly *α*-Angle and MA, provide valuable predictive information. These findings support the integration of individualized antiplatelet strategies and TEG-guided monitoring to optimize outcomes in OVAS patients.

## Introduction

Stroke is the second leading cause of mortality worldwide (1). Unlike ischemic strokes in the anterior circulation, those occurring in the posterior circulation often received much less attention (2). Crucially, one-fifth of strokes occur in the posterior circulation (3, 4). Posterior circulation strokes are frequently associated with worse clinical outcomes, the higher rates of disability and mortality compared to anterior circulation events impose a substantial socioeconomic burden on patients and healthcare systems (5).

Approximately 20% posterior circulation strokes stem from atherosclerotic ostial vertebral stenosis (OVAS) (6). The first-line treatment for OVAS is antiplatelet therapy along with management of major risk factors, however, the recurrence rate remains unacceptably high in patients with OVAS. For patients treated with medical therapy alone, recurrence risk rises notably in the first few weeks following symptom presentation, with an annual stroke rate reaching 10–15% (5). Recently, with the improvement of neurointerventional techniques, endovascular therapy has become one of the key options for OVAS, characterized by minimal invasion and sustained effect. Previous studies have shown that stenting treatment for OVAS significantly reduces recurrence rates and improves prognosis. However, complications caused by in-stent restenosis (ISR) limit the application of stenting in the treatment of OVAS. Over 20 to 30% patients received stenting treatment experience restenosis within six months, representing the most common and severe complication for stenting treatment, particularly when bare metal stents (BMSs) are utilized (7–9).

Emerging evidence indicates that recurrent symptoms following BMSs implantation in the extracranial vertebral artery are predominantly linked to ISR (10), often necessitating secondary interventions that further predispose patients to hemorrhagic or thrombotic risks. Recently, utilization of drug-eluting stents (DESs) for treatment of OVAS have gained significant clinical traction. DESs are characterized by eluted drugs that inhibit vascular endothelial cell proliferation on the surface or inside of stents. Release of drugs can prevent in-stent restenosis of OVAS, suppress intravascular thrombosis and reduce stroke recurrence (11, 12). Compared with BMSs, DESs exhibit mid- and long-term superiority. However, few studies have focused on the application of DESs in OVAS to date. Among the few studies, most are descriptive studies that only focus on the safety and validity of DES implantation in OVAS and lack rigorous longitudinal follow-up and mechanistic analysis (13–18).

Optimizing post-procedural outcomes also necessitates robust antithrombotic management. Current protocols mandate dual antiplatelet therapy (DAPT) for at least 3 to 6 months post-stenting. However, resistance to antiplatelet drugs, especially clopidogrel, remains a prevalent clinical challenge (14). Previous studies have suggested that antiplatelet drug resistance may contribute to the recurrence of cerebral infarction (14). To date, few studies have investigated the relationships between variants of antiplatelet-related genes and stent restenosis, which are included in this study.

Therefore, this single-center retrospective study sought to integrate multi-domain data and analyze one-year prognosis, postoperative restenosis and independent risk factor of restenosis in patients with OVAS who underwent DESs implantation.

## Materials and methods

### Study design and patient enrollment

This retrospective study was carried out between January 2022 to July 2025 in Cerebrovascular Stroke Center, Nanjing Brain Hospital, PR China. A total of 446 patients were screened. Patients who met the following criteria were enrolled: (1) people aged 18 to 80 years; (2) symptomatic stenosis ≥50% or asymptomatic stenosis ≥70% according to angiographic findings; (3) patients who experienced posterior circulation transient ischemic attack (TIA), vertigo or non-disabling stroke and other ischemic symptoms during the past 3 months; (4) Modified Rankin Scale ≤3 and National Institutes of Health Stroke Scale ≤6; (5) patients had received standard antiplatelet and statin therapy, but there were recurrent symptoms. Exclusion criteria are as following (19): (1) intracranial hemorrhage or active gastric ulcer in the past 3 months; (2) culprit artery with severe tortuosity, calcification or malformation; severe calcification was defined as circumferential calcification or heavy calcification precluding adequate stent expansion which determined by pre-procedural CTA or DSA. (3) cardioembolism caused by atrial fibrillation or valvular heart disease that need long-term anticoagulation; (4) advanced malignancy or severe organ failure; (5) allergy to contrast agent, aspirin or clopidogrel. ISR diagnostic criteria: in-stent or peri-stent (within 5 mm of either stent end) angiographic luminal narrowing >50% on evaluable CTA images or end diastolic velocity (EDV) ≥ 45 cm/s, peak systolic velocity (PSV) ≥ 150 cm/s by colored Doppler ultrasound (CDUS) (20). Stent tortuosity was defined as vessel undulation that referred to S-, C-, a-, x-, or Z-shaped elongation, kinking, or coiling which assessed by two independent neuroradiologists through DSA (21, 22). This definition was uniformly applied across all cases. All interventionalists (*n* = 6) participated in a standardized training session before the study period to ensure consistent assessment.

Thromboelastography (TEG) measurements were performed one month after surgery for all enrolled patients. TEG was conducted with either non-citrated or citrated whole blood. Citrated tubes were left at room temperature for 30 min before test, while non-citrated samples must be loaded into the analyzer within 4–6 min of collection to prevent spontaneous clotting. Calcium chloride was added to the cup to trigger coagulation. A torsion-wire-held pin sit in the blood while the cup rotated 4.45° every 10s. As clot strength built, shear forces transmitted the cup’s motion to the pin, and an electromechanical transducer converted the pin’s displacement into TEG trace (23).

Pharmacogenomic analyses for both clopidogrel and aspirin were carried out by a specialized, certified third-party laboratory (Beijing Huaxia Gene Technology Co., Ltd.) with mass spectrometry. Clopidogrel CYP2C19 metabolic phenotypes are classified as Rapid Metabolizers (NM): *1/*1,*1/*17, and *17/*17, Intermediate Metabolizers (IM): *1/*2,*1/*3, *2/*17,*3/*17, Poor Metabolizers (PM): *2/*2,*2/*3,3/*3.

### Stent implantation procedure

The imaging data of all patients that underwent stent implantation were evaluated independently by two physicians with over five years of experience in neuroradiology. All patients needed to take aspirin 100 mg/d and clopidogrel 75 mg/d for at least 3 to 5 days as a preoperative preparation. Femoral artery was chosen as the access route. The sizes of stents were usually decided by physicians based on culprit vessel diameter. Unfractionated heparin was injected via vein 70-100 IU/kg before procedure to achieve systemic heparinization, then, based on activated clotting time (ACT) further heparin was added to maintain ACT 250–350 s. An 8-F guiding catheter was delivered to the subclavian artery at the origin of the vertebral artery (left or right, depending on the lesion side), subsequently, a 0.014-inch micro guidewire crossed the culprit vessel lesion gently with the help of road-map imaging. Semi-compliant balloon (2.0 × 20 mm or 3.0 × 20 mm or 4.0 × 20 mm) was used to conduct low-pressure pre-dilatation to prevent dissection, then slowly withdrawn after the implantation of Maurora Stent (Alain Medical, Beijing, China). Immediate angiography was conducted to assess residual stenosis after stent implantation. All patients were required to take aspirin 100 mg/d and clopidogrel 75 mg/d for 6 months, and then switch to monotherapy for lifelong.

### Follow-up

At admission and discharge, National Institutes of Health Stroke Scale (NIHSS), modified Rankin Scale (mRS) and Activities of Daily Living (ADL) were conducted for all patients. During hospitalization, clinical and angiographic data, perioperative complications, surgical success rate, postoperative adverse events and follow-up information were collected for all patients.

Vertebral artery duplex ultrasounds were conducted to assess blood flow in the stent at admission, one, six and twelve months after stent implantation, respectively. The final follow-up data were used to assess ISR. If symptomatic stenosis occurred or duplex ultrasound suggested possible ISR, further computed tomography angiography (CTA) or digital subtraction angiography (DSA) were advised. ISR was defined as luminal diameter stenosis ≥50% after stent implantation by angiographic evidence (19, 24). If residual stenosis of the culprit vessel lesion was >50% or ISR needed retreatment, these cases would be considered stenting failures.

### Statistical methods

All statistical analyses were performed using SPSS version 27.0. Categorical variables are expressed as frequencies and percentages, and between-group comparisons were made using the χ^2^ test. Normally distributed continuous data are presented as mean ± standard deviation (SD) and compared between the two groups with an independent-samples t test. Non-normally distributed continuous variables are described as median and interquartile range (IQR) and compared with the Mann–Whitney U test. A binary logistic regression model was used to identify independent risk factors for in-stent restenosis, and the predictive performance was assessed by the area under the receiver operating characteristic (ROC) curve. All tests were two-sided, and *p* < 0.05 was considered statistically significant.

## Results

### Patient characteristics

**Figure 1:**
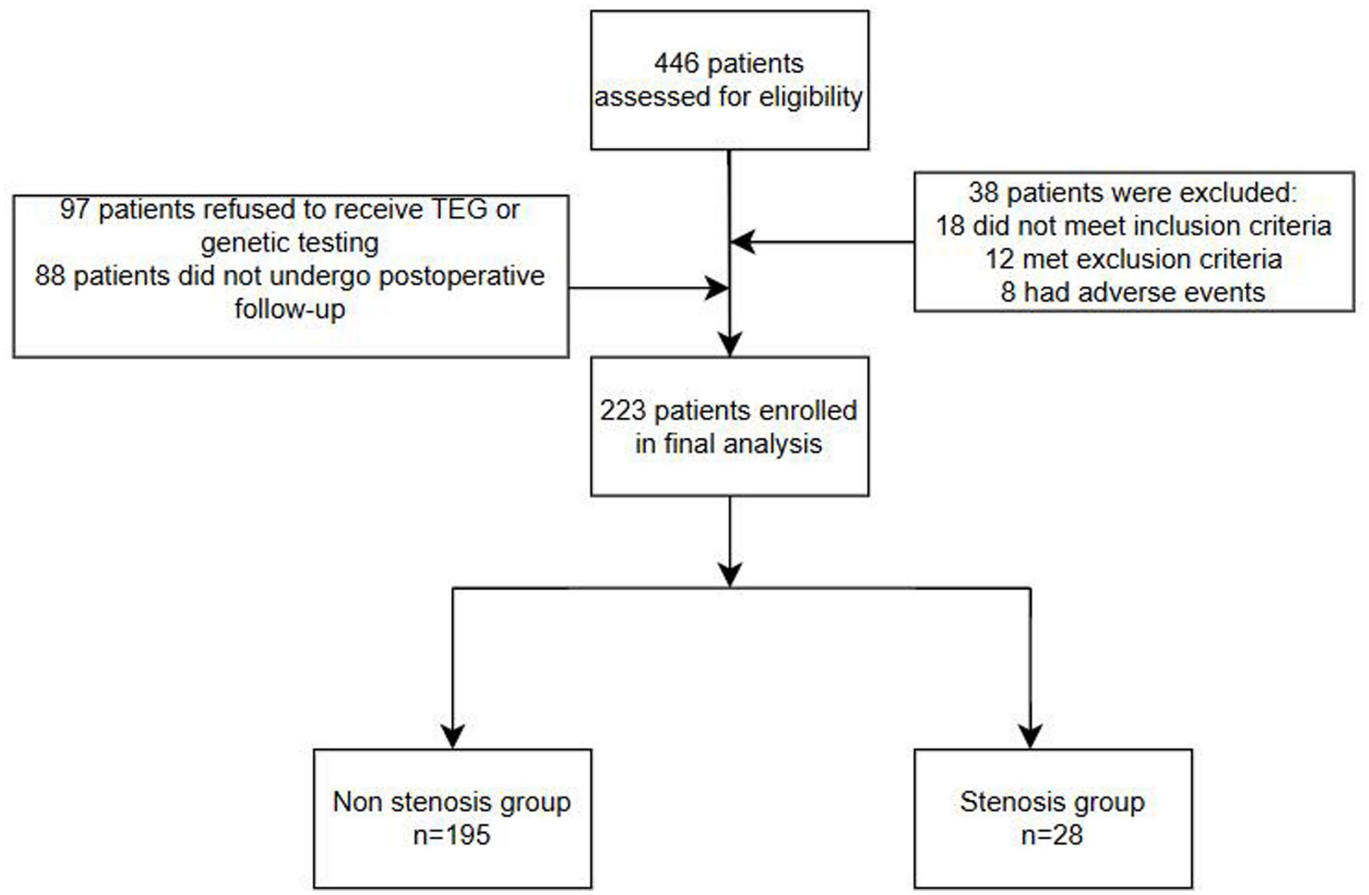
Patient enrollment flowchart.

This trial involved 446 patients, for various reasons 223 were enrolled in the final analysis (Figure 1). Of the 223 patients analyzed, 195 had patent stents and 28 had in-stent restenosis. Among the 28 ISR cases, 3 cases were detected at 6 months, and 25 cases were detected at 12 months, moreover, all patients were asymptomatic. There were 8 adverse events including 2 cases of hyperperfusion syndrome, 2 cases of intracranial subarachnoid hemorrhage (*n* = 2), 1 case of death during follow-up, and 3 case of discontinuation of antiplatelet therapy due to other surgical procedures.

The restenosis group was older (71.9 ± 8.3 vs. 67.2 ± 8.5 years, *p* = 0.007) and had significantly higher rates of hypertension (89.3% vs. 66.7%, *p* = 0.015) and smoking (60.7% vs. 32.8%, *p* = 0.004). Stent tortuosity was also more frequent in the restenosis group (53.6% vs. 11.8%, *p* = 0.001). No significant differences were observed for gender, diabetes, atrial fibrillation, hyperlipidemia, alcohol use, prior coronary heart disease, stroke or TIA, or for stent diameter, length, or left-sided lesion location (*p* > 0.05) (Table 1).

**Table 1:** Baseline characteristic of the study patients.

Characteristics	Non-stenosis (*n* = 195)	Stenosis (*n* = 28)	*p* value
Age, mean ± SD, years	67.160 ± 8.519	71.860 ± 8.312	0.007
Gender, male, *n*(%)	146 (74.87%)	19 (67.86%)	0.429
Hypertension, *n*(%)	130 (66.67%)	25 (89.29%)	0.015
Smoking, *n*(%)	64 (32.82%)	17 (60.71%)	0.004
Alcohol, *n*(%)	39 (20.00%)	5 (17.86%)	0.790
Diabetes, *n*(%)	69 (35.38%)	7 (25.00%)	0.278
Atrial fibrillation, *n*(%)	4 (2.05%)	1 (3.57%)	0.492
Hyperlipidmia, *n*(%)	12 (6.15%)	1 (3.57%)	0.909
Coronary heart disease, *n*(%)	21 (10.77%)	5 (17.86%)	0.437
Stroke, *n*(%)	93 (47.69%)	14 (50.00%)	0.819
TIA, *n*(%)	53 (27.18%)	4 (14.29%)	0.144
Stent diameter (mm)	4.003 ± 0.747	3.804 ± 0.712	0.186
Stent length (mm)	15.195 ± 2.945	14.714 ± 3.090	0.423
Stent tortuosity, *n*(%)	23 (11.79%)	15 (53.57%)	0.001
Left side, *n*(%)	84 (43.08%)	17 (60.71%)	0.080

TIA: Transient Ischemic Attack.

Technical success was achieved in all patients, with a total of 467 drug-eluting stents (DESs) successfully implanted. There were no intraoperative complications such as embolism, cerebral hemorrhage, arterial dissection or contrast extravasation. Only one patient experienced a sharp drop blood pressure during the procedure, but got fully recovery after medication intervention. Ten patients received bilateral vertebral arteries stenting and seven patients received ipsilateral vertebral artery stenting with two stents implanted. Immediate DSA after stenting showed residual stenosis was <30% and blood flow was restored.

Post-procedurally, patients adhered to a 6-month regimen of dual antiplatelet therapy (DAPT), followed by lifelong antiplatelet monotherapy. 128 patients were evaluated with CDUS, 25 were evaluated with CDUS and CTA, 2 were evaluated with MRA and CDUS and only 4 were evaluated with DSA. ISR was found in 28 cases during the follow-up, indicating a rate of 12.56%.

The distribution of genotypes and alleles of GPIIIa, PTGS1 and PEAR1 genes in non-stenosis group (*n* = 121) and stenosis group (*n* = 17) were summarized in Table 2. For the GPIIIa gene, the TT genotype and T allele were predominant in both groups. For the PTGS1 gene, only the AA genotype and A allele were detected in both groups. For the PEAR1 gene, AA/AG/GG genotypes and A/G alleles were detected in both groups. There was no significant difference between the two groups for any of these genes (*p* > 0.05). Clopidogrel metabolic types were classified according to the corresponding CYP2C19 genotypes (Table 3). No rapid metabolizers were detected in either group. There were no differences in ultrarapid, intermediate, normal metabolic types between the two groups (*p* > 0.05). However, poor metabolizers were more prevalent in the stenosis group compared with the non-stenosis group, with a significant difference (X^2^ = 4.387, *p* = 0.036), indicating an association between CYP2C19 poor metabolism and in-stent stenosis.

**Table 2:** Genotype distribution and allele comparison.

Genes	Genotypes	Genetic marker	Non-stenosis (*n* = 121)	Stenosis (*n* = 17)	*p* value
GPIIIa	Genotypes	CT	1	0	1.000
		TT	120	17	0.969
	Allele	C	1	0	1.000
		T	241	34	0.978
PTGS1	Genotypes	AA	121	17	0.846
	Allele	A	242	34	0.783
PEAR1	Genotypes	AA	21	2	0.779
		AG	57	11	0.358
		GG	43	4	0.353
	Allele	A	99	15	0.894
		G	143	19	0.658

**Table 3:** Effects of CYP2C19 gene polymorphisms on Clopidogrel metabolism.

Metabolic type	Stenosis (*n* = 18)	Non-stenosis (*n* = 121)	X^2^	*p* value
Ultrarapid metabolizer	0	9	0.495	0.467
Rapid metabolizer	0	0	0	0
Intermediate metabolizer	9	57	0.819	0.053
Normal metabolizer	3	41	0.143	2.147
Poor metabolizer	6	14	0.036	4.387

TEG parameters of the two groups were showed in Table 4. R time was significantly shorter in stenosis group (*p* < 0.001). Clot formation rate and maximum clot strength were significantly increased in stenosis group, which was reflected by higher *α*-Angle and MA value (*p* < 0.001). Fibrinolysis after 30 min (LY30), ADP% inhibition and AA% inhibition were comparable between the two groups (*p* > 0.05). There was no significant difference between the two groups for K time, LY30, ADP% inhibition and AA% inhibition (*p* > 0.05), yet R time, *α*-Angle and MA value correlated with in-stent stenosis (*p* < 0.001).

**Table 4:** Comparison of TEG between the two patient groups.

Group	Non-stenosis	Stenosis	t/Z value	*p* value
R time (min)	5.968 ± 1.217	5.281 ± 0.797	4.758	<0.001
K time (min)	1.496 ± 0.514	1.481 ± 0.327	−0.101	0.920
α-Angle (°)	64.156 ± 6.183	69.731 ± 3.323	−5.304	<0.001
MA (mm)	61.702 ± 5.095	66.944 ± 4.248	−5.407	<0.001
LY30 (%)	0.202 ± 0.499	0.100 ± 0.000	−1.385	0.166
ADP-% inhibition	65.243 ± 20.308	69.774 ± 25.182	−0.505	0.614
AA-% inhibition	93.822 ± 14.462	91.494 ± 16.665	−0.363	0.716

According to the results of multi-factor correlation, hypertension, smoking, age, stent tortuosity and poor clopidogrel metabolic type, TEG R time, *α*-Angle, MA value were correlated with ISR with univariable analysis (*p* < 0.05). Using in-stent stenosis as the dependent variable, those variables with *p* < 0.05 in univariable analysis were used as independent variables for the multivariate analysis. Results indicated only α-Angle and MA value were independent risk factors for postoperative ISR in patients with OVAS (*p* < 0.05) (Table 5).

**Table 5:** Multivariate analysis of risk factors for in-stent restenosis.

Factor	B	SE	OR (95%CI)	*p* value
Smoking	−0.673	0.805	0.510 (0.105–2.473)	0.403
Poor metabolizer	−0.195	0.840	0.823 (0.159–4.264)	0.816
Stent tortuosity	−1.297	0.841	0.273 (0.053–1.422)	0.123
Age	0.071	0.046	1.074 (0.981–1.175)	0.124
Hypertension	−1.005	1.313	0.366 (0.028–4.803)	0.444
R time	0.035	0.358	1.036 (0.514–2.088)	0.922
α-Angle	0.195	0.087	1.215 (1.025–1.441)	0.025
MA	0.222	0.101	1.249 (1.025–1.522)	0.027

**Figure 2:**
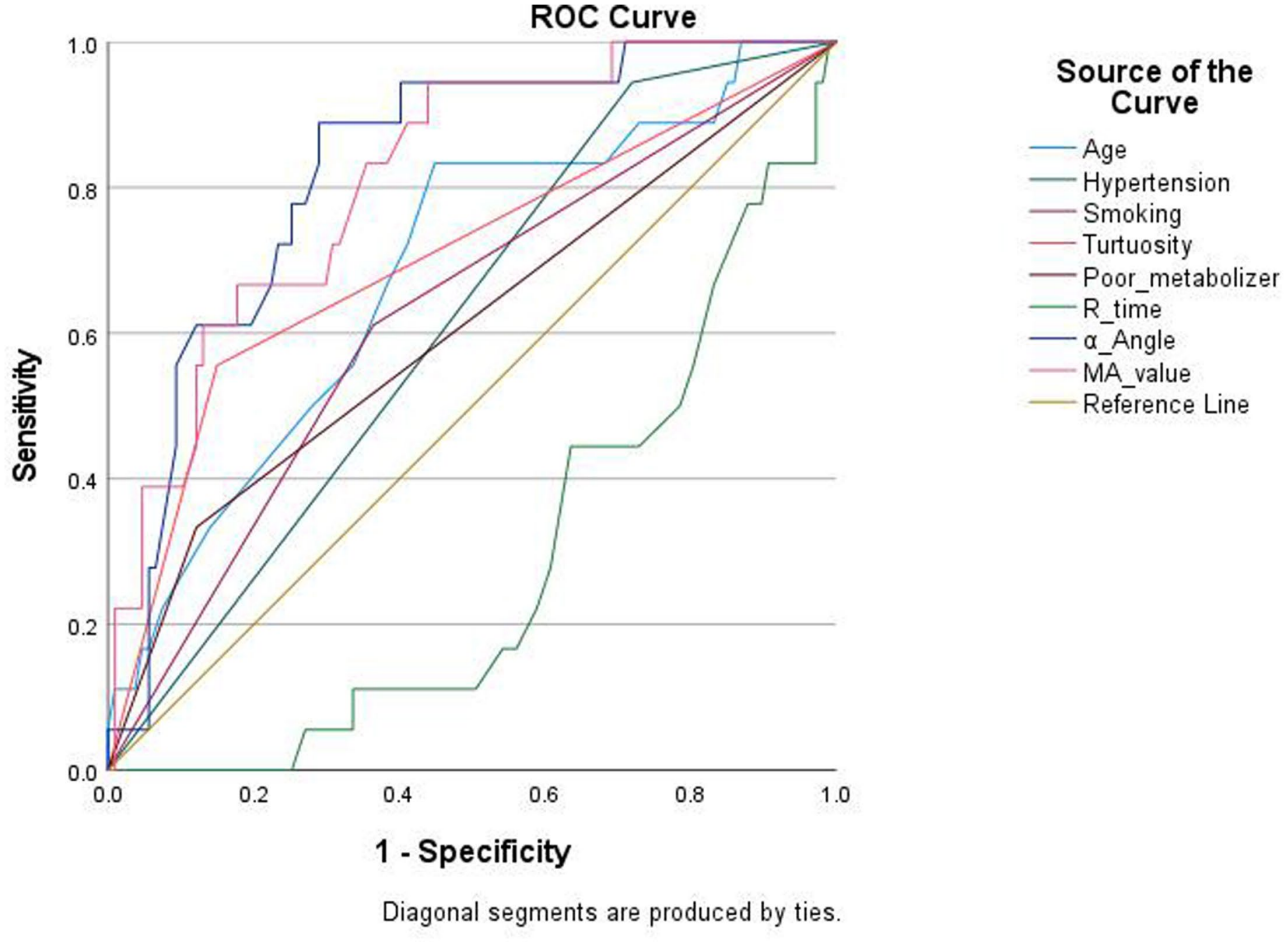
ROC curve for predicting in-stent restenosis.

Figure 2 showed receiver-operating-characteristic (ROC) curves of these 8 independent variables for ISR. Among clinical indicators, stent tortuosity achieved the highest AUC (0.703), followed by age (0.686) and smoking history (0.623). Hypertension and poor metabolic status only showed modest predictive value. TEG parameters demonstrated better performance compared with clinical indicators, as *α*-Angle achieved an AUC of 0.826 with a sensitivity of 80.4% and a specificity of 75.9%, and MA value achieved an AUC of 0.814 with a sensitivity of 78.6% and a specificity of 73.8%, indicating excellent ability to predict ISR in patients with OVAS. In contrast, R time showed no predictive value.

**Figure 3:**
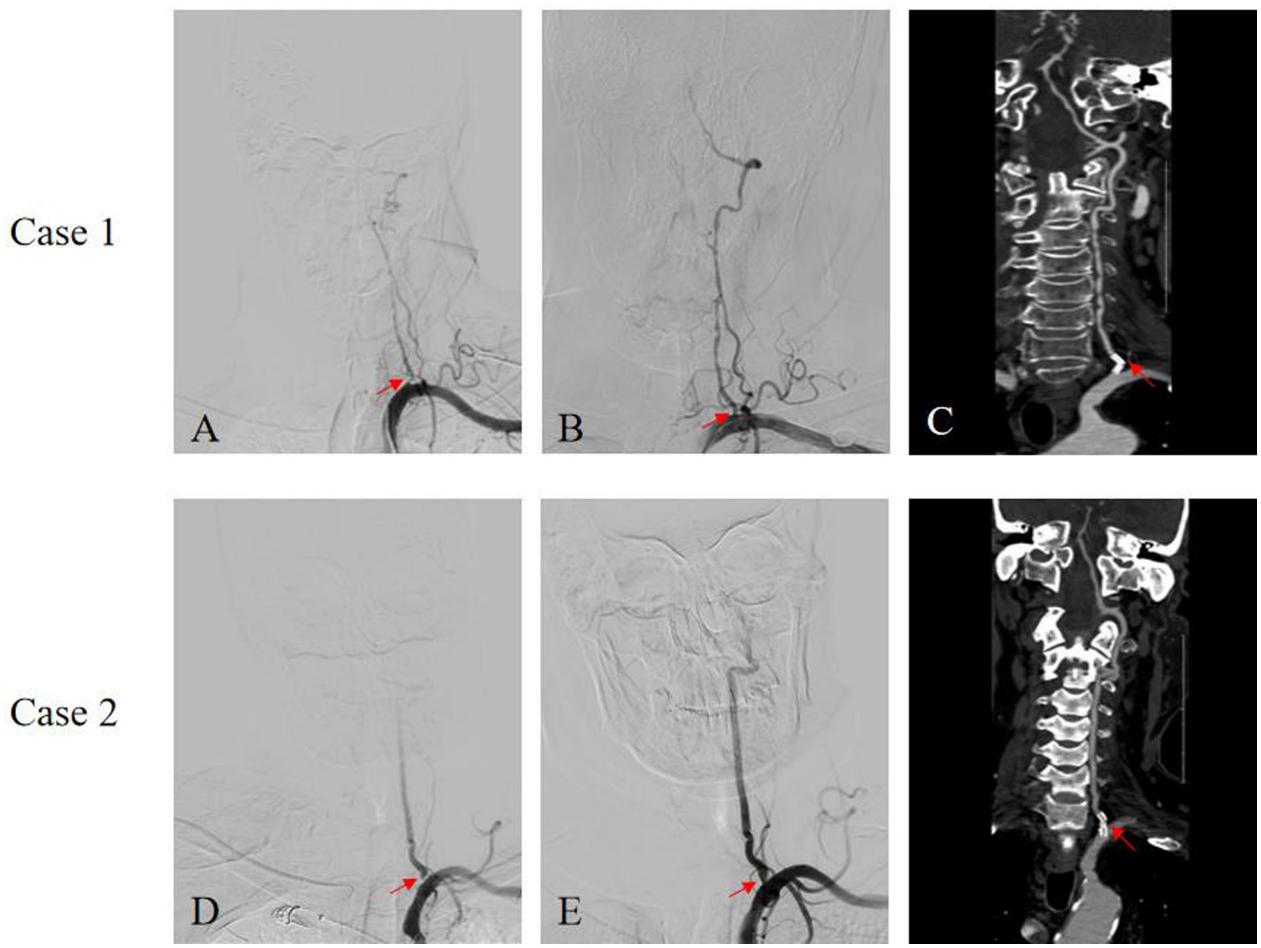
Stent fracture in two cases. **(A)** Pre-operative (Case 1); **(B)** Post-stenting (Case 1); **(C)** Six-month CTA(Case 1); **(D)** Pre-operative (Case 2); **(E)** Post-stenting (Case 2); **(F)** Six-month CTA (Case 2).

Descriptions of two distinct complications of vertebral artery ostial stenting are shown in Figure 3:

Case 1: One patient experienced a recurrence of posterior circulation ischemic symptoms several months after stent fracture. Follow-up angiography revealed significant in-stent restenosis within the implanted stent.

Case 2: One patient was found to have stent fracture during a scheduled follow-up examination. CTA showed a clear break in the stent strut at the site of maximal mechanical stress.

## Discussion

In the present study, we observed a one-year ISR incidence of 12.56% following DES implantation. This finding aligns with the results reported by Hajiyev et al. (25), which compared the efficacy of DES and BMS in symptomatic intracranial and vertebral artery stenosis.

The DES used in our study is the first-generation DES dedicated to extracranial vertebral artery stenosis, which was launched in July 2020. The stent was composed of L605 cobalt-chromium alloy, which provided excellent flexibility and radial strength (12). The stent was coated with rapamycin released consistently to prevent neointimal hyperplasia by suppressing migration and proliferation of endothelial cells and smooth muscle cells (7, 12). Thus, substantial data has supported that endovascular therapy with DESs exhibited higher success rate, lower incidence of major perioperative complications and significantly lower ISR than in previous studies with BMSs (7).

Importantly, our results underscore that ISR is not a stochastic event but is closely associated with high platelet reactivity (HPR) induced by poor metabolism of clopidogrel and higher *α*-Angle and MA values. Mechanistically, poor clopidogrel metabolizers exhibit impaired CYP2C19-mediated oxidative conversion, leading to a deficiency in thiol-containing active metabolites. This deficiency results in suboptimal irreversible blockade of the P2Y12 receptor thereby facilitating paradoxical platelet aggregation (26). Moreover, macrophages could regulate local inflammation and drive vascular smooth muscle cell (VSMC) proliferation and intimal hyperplasia by secreting a variety of cytokines, including TNF-*α* and IL-1β, which might contribute to the development of ISR (27).

TEG serves as a robust diagnostic platform for the dynamic and comprehensive monitoring of whole-blood coagulation. Currently, it is widely utilized to titrate anticoagulant and antiplatelet therapies and to preempt thrombotic events. Our findings revealed that patients with ISR exhibited significantly elevated MA values and larger *α*-angles, indicating HPR and clopidogrel resistance, a higher MA value indicates hyperfunction of platelets, which may increase the thrombus risk in patients, especially in those with stent restenosis. Elevated MA value may indicate platelet activation after stent implantation, platelets may be activated, leading to inflammation statue of vascular endothelium caused by stent stimulation.

This inflammatory milieu likely elevates fibrinogen levels, thereby increasing clot strength. As for the *α*-angle that reflects the speed of fibrin formation, it is closely related to the K value. In our results, an increase in this value may indicate accelerated fibrin formation and increased blood coagulability. In patients with stent restenosis, an elevated α angle may also be due to the inflammatory response after stent implantation. Local inflammation after stent implantation may lead to increased fibrinogen levels, accelerating fibrin formation. When the body is in a hypercoagulable state, the risk of endothelial damage and thrombosis increases, leading to restenosis. These patients also show insensitivity to clopidogrel, which indicated ISR is relative to a higher risk of thrombosis. Other TEG parameters show no correlation with ISR, this may be explained by the fact that ISR is a multifactorial disease (28), and individual parameters may be inadequate to predict correlation with ISR, as TEG only assesses platelet function.

In clinical practice, CYP2C19 genotyping is a cornerstone for predicting the therapeutic efficacy of clopidogrel. CYP2C19 plays an important role in helping clopidogrel oxidative metabolism become an active metabolite with antiplatelet activity in the hepatic cytochrome P450 system (29). CYP2C19 genetic polymorphism is thought an independent risk factor that can decide antiplatelet efficacy based on clopidogrel (30–32). Clopidogrel needs to be metabolized into its active form by the liver CYP2C19 enzyme. If a patient carries a loss-of-function allele (such as CYP2C192 or 3), the production of the active metabolite is reduced, leading to a weakened antiplatelet effect. Our data reinforce the consensus that genetic polymorphism may lead to insufficient drug inhibition of platelet aggregation, increased stent thrombosis, and ultimately stent restenosis (33). It has been established that the existence of LOF allele can lead to loss of clopidogrel function, this may associate with ISR postoperatively (34, 35). This pharmacogenomic deficit contributes to an increased incidence of stent thrombosis and subsequent ISR. Consistent with previous literature, our results suggest that the presence of LOF alleles is a major determinant of post-procedural restenosis in OVAS, emphasizing the necessity of integrating genetic testing to tailor individualized antiplatelet protocols.

The ROC curve analysis in this study demonstrated that *α*-Angle achieved an AUC of 0.826 with a sensitivity of 80.4% and a specificity of 75.9% and MA value achieved an AUC of 0.814 with a sensitivity of 78.6% and a specificity of 73.8%. These metrics indicate an excellent capacity for ISR risk stratification. MA value and α-Angle which closely associated with ISR are key parameters for evaluating platelet function during thrombosis. Thus TEG can serve as an effective clinical tool to identify ISR. It enables clinicians to identify high-risk OVAS patients at an early stage, allowing for timely pharmacological adjustments, such as the transition to more potent P2Y12 inhibitors or the intensification of monitoring.

As summarized in Table 1, advanced age, tobacco use, and stent tortuosity were significant predispositions for ISR. Smoking can promote inflammatory cascades through mitochondrial oxidative stress response and oxidative endothelial damage, accelerating neointimal hyperplasia and increasing the risk of ISR (36). Ageing reduces endothelial regenerative ability, shortens telomeres, and increases collagen and calcium deposition in the vessel wall which creates an environment that favors fibrotic remodeling, elastic recoil and neointimal hyperplasia around stent (37, 38). From a biomechanical perspective, stent tortuosity creates regions of low wall shear stress and suboptimal wall apposition, which triggers focal platelet deposition and subsequent smooth muscle cell hyperplasia, ultimately culminating in ISR (39).

Our study showed a slightly higher in-stent restenosis rate compared to other studies (13, 14, 18). One reason is that most prior studies did not include color-Doppler ultrasound as a screening criterion, and Doppler findings can be highly operator-dependent. Notably, the majority of ISR cases in our cohort were asymptomatic; the single symptomatic case is detailed in Figure 3. In these patients, the Doppler velocities failed to show a marked increase. As for the two cases of stent fracture showed in Figure 3, the likely causes of stent fracture may due to the repetitive bending of the vertebral artery during respiration and neck rotation, which generates cyclic flexural stress, representing the most important external cause of fatigue fracture. Given that drug-eluting stents are relatively flexible, they may be more susceptible to structural failure when subjected to complex shear forces and sustained high-pressure hemodynamics, which increase the radial alternating loads on the stent struts.

Several limitations of this study warrant consideration. First, it is a single-center observational investigation with a relatively small sample size and a short follow-up time (one year), which might be insufficient to capture very late in-stent restenosis events. Second, a degree of selection bias may exist due to incomplete data or loss to follow-up in a subset of patients. Onsequently, large-scale, multicenter, prospective randomized controlled trials are necessitated to validate these preliminary findings and establish more robust clinical correlations.

In conclusion, this study shows that DES restenosis results from the combined action of various factors, including age, hypertension, stent tortuosity, smoking, poor clopidogrel metabolic status, larger *α*-angle and MA value and shorter R time. These findings advocate for a comprehensive, multi-domain evaluative strategy to facilitate personalized antiplatelet therapy and intensive monitoring, which may ultimately optimize long-term outcomes and curb the development of ISR in the OVAS patient population.

## Data Availability

The raw data supporting the conclusions of this article contains privacy-sensitive information (including patient names and genetic data) that is not suitable for public sharing due to ethical and privacy restrictions. Qualified researchers may request access to the anonymized data by contacting the corresponding author at the following email address: qiufeng11@sina.com.
